# Comparisons of Prognosis between Surgically and Clinically Diagnosed Idiopathic Pulmonary Fibrosis Using Gap Model: A Korean National Cohort Study

**DOI:** 10.1097/MD.0000000000003105

**Published:** 2016-03-18

**Authors:** Sang Hoon Lee, Song Yee Kim, Dong Soon Kim, Young Whan Kim, Man Pyo Chung, Soo Taek Uh, Choon Sik Park, Sung Hwan Jeong, Yong Bum Park, Hong Lyeol Lee, Jong Wook Shin, Eun Joo Lee, Jin Hwa Lee, Yangin Jegal, Hyun Kyung Lee, Yong Hyun Kim, Jin Woo Song, Moo Suk Park

**Affiliations:** From the Department of Internal Medicine, Division of Pulmonology, Severance Hospital, Institute of Chest Diseases, Yonsei University College of Medicine, Seoul, Korea (SHL, SYK, MSP); Division of Pulmonary and Critical Care Medicine, University of Ulsan College of Medicine, Asan Medical Center (DSK, JWS); Department of Internal Medicine and Lung Institute, Division of Pulmonary and Critical Care Medicine, Seoul National University College of Medicine (YWK); Division of Pulmonary and Critical Care Medicine, Samsung Medical Center, Sungkyunkwan University School of Medicine (MPC); Department of Internal Medicine, Division of Allergy and Respiratory Medicine, Soonchunhyang University Seoul Hospital (STU); Department of Internal Medicine, Division of Allergy and Respiratory Medicine, Soonchunhyang University Bucheon Hospital (CSP); Department of Internal Medicine, Division of Pulmonology, Gachon University Gil Medical Center (SHJ); Department of Internal Medicine, Division of Pulmonary, Allergy & Critical Care Medicine, Hallym University Kangdong Sacred Heart Hospital (YBP); Department of Internal Medicine, Pulmonary Division, Inha University Hospital (HLL); Department of Internal medicine, Division of Pulmonary Medicine, Chung Ang University College of Medicine (JWS); Division of Respiratory and Critical Care Medicine, Department of Internal Medicine, Korea University Anam Hospital, Korea University College of Medicine (EJL); Department of Internal Medicine, Ewha Womans University School of Medicine, Ewha Medical Research Institute (JHL); Department of Internal Medicine, Division of Pulmonary Medicine, Ulsan University Hospital, University of Ulsan College of Medicine (YJ); Department of Internal Medicine, Division of Critical Care and Pulmonary Medicine, Inje University Pusan Paik Hospital (HKL); and Department of Internal Medicine, Division of Allergy and Pulmonology, Bucheon St. Mary's Hospital, The Catholic University of Korea School of Medicine (YHK), Bucheon, Korea.

## Abstract

Although a multidisciplinary approach has become an important criterion for an idiopathic pulmonary fibrosis (IPF) diagnosis, lung biopsies remain crucial. However, the prognosis of patients with surgically diagnosed IPF (sIPF) is uncertain. We aimed to investigate the prognosis of patients with clinically diagnosed IPF (cIPF) and sIPF.

In this retrospective observational study, the Korean Interstitial Lung Disease Study Group conducted a national survey to evaluate the clinical, physiological, radiological, and survival characteristics of patients with IPF from January 1, 2003 to December 31, 2007. Patients were recruited from 54 universities and teaching hospitals across the Republic of Korea. IPF diagnoses were established according to the 2002 American Thoracic Society (ATS)/European Respiratory Society criteria (ERS) guideline. A total of 1685 patients with IPF (1027 cIPF and 658 sIPF) were enrolled.

Patients with sIPF were significantly younger, predominantly female, and nonsmokers (all *P* < 0.001). sIPF group had significantly better initial pulmonary function. The proportion of computed tomography-based honeycomb findings of patients with cIPF was higher than in those with sIPF (*P* < 0.001). A Kaplan-Meier analysis showed that the sIPF group had a better prognosis (*P* = 0.001). A survival analysis showed that age, pulmonary function parameters, pulmonary oxygen tension, honeycombing change, and combined lung cancer had a significant influence on patient prognosis. However, there was no significant difference in prognosis between the cIPF and sIPF groups after adjusting for GAP (gender, age, physiology) stage.

The patients with sIPF had better clinical features than those with cIPF. However, after adjusting for GAP stage, the sIPF group showed similar prognoses as the cIPF group. This study showed that after adjusting for GAP stage, the prognosis of patients with IPF is the same regardless of the diagnostic method used.

## Abstract 2

Supplemental Digital Content is available in the text

## INTRODUCTION

Idiopathic pulmonary fibrosis (IPF) is the most common form of idiopathic interstitial pneumonias.^1^ IPF is defined as a specific form of progressive and chronic fibrosing interstitial pneumonia without a definite cause. It occurs primarily in older patients, especially in the sixth and seventh decades, and is limited to the lungs.^2,3^ It is also associated with increasing respiratory symptoms and irreversible respiratory failure.^3^

Although IPF could be diagnosed clinically in the absence of a surgical lung biopsy, it was recognised as a distinct clinical entity that was associated with the histologic pattern of usual interstitial pneumonia (UIP).^2,4^ In the last few years, the paradigm of diagnosis of IPF has gradually changed from a situation in which biopsy was the criterion standard to a complex situation in which the multidisciplinary approach was necessary. Such approach encompasses clinical, radiological, and pathologic data.^2,5–7^ However, surgical biopsy is still needed for IPF diagnoses because there are cases that cannot be diagnosed without the histologic pattern.

It is well known that the median survival of patients with IPF is <3 years.^8,9^ To provide precise prognostic information and timely treatment to these patients, many predictive models have been investigated. Previous studies have shown that older age at diagnosis, male sex, decreased pulmonary function, and impaired exercise capacity predict a worse outcome in patients with IPF.^8–15^ However, the prognosis of patients with IPF with a nontypical computed tomography pattern, who were eventually diagnosed by surgical lung biopsy examination, was largely unknown. In 2012, Ley et al^16^ reported a simple-to-use GAP (gender, age, physiology) model for predicting IPF mortality, which is a scoring and staging system like the one for lung cancer. This novel model consists of 4 clinical variables: gender (G), age (A), and 2 pulmonary physiology parameters (P, FVC, and DL_CO_). Each variable was assigned 1 to 3 points and then added for staging; stage I (0–3 points), stage II (4–5 points), and stage III (6–8 points). The purpose of this study is to evaluate whether clinically diagnosed IPF (cIPF) and surgically diagnosed IPF (sIPF) have different characteristics. Furthermore, we aimed to evaluate the role and effect of surgical biopsy in predicting prognosis in conjunction with the GAP staging system.

## PATIENTS AND METHODS

### Patient Selection

**FIGURE 1:**
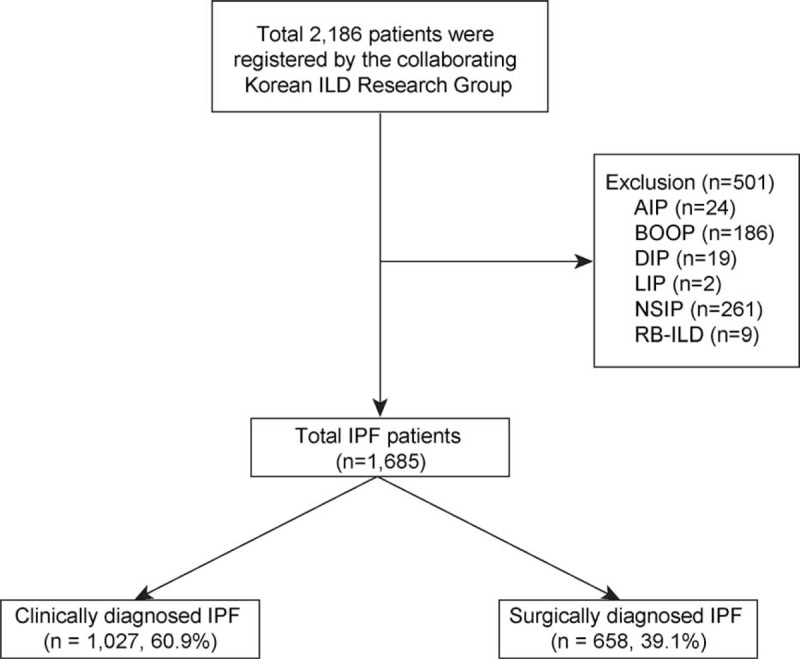
Flow chart of inclusion and exclusion of patients in the study. A total of 2186 patients were registered at 54 centers in Korea and of the total, 1685 patients were divided into clinically diagnosed IPF (cIPF, n = 1027) or surgically diagnosed IPF (sIPF, n = 658). AIP = acute interstitial pneumonia, BOOP = bronchiolitis obliterans organizing pneumonia, DIP = desquamative interstitial pneumonia, ILD = interstitial lung disease, LIP = lymphocytic interstitial pneumonia, NSIP = nonspecific interstitial pneumonia, RB-ILD = respiratory bronchiolitis-associated interstitial lung disease.

The Scientific Committee in the Korean Academy of Tuberculosis and Respiratory Diseases, comprising 54 universities and teaching hospitals, contacted pulmonary specialists (n = 82) to identify patients with IPF. Newly diagnosed adult IPF (≥30 years) patients were enrolled between 2003 and 2007. Patients with a defined connective tissue disease, left ventricular failure, or a history of ingestion of a drug or an agent known to cause pulmonary fibrosis were excluded from the study. In total, 2186 patients were initially registered. Of these patients, the following other forms of idiopathic interstitial pneumonias (n = 501) were also excluded in this study (Figure 1): acute interstitial pneumonia (AIP), bronchiolitis obliterans organizing pneumonia (BOOP), desquamative interstitial pneumonia (DIP), lymphocytic interstitial pneumonia (LIP), nonspecific interstitial pneumonia (NSIP), and respiratory bronchiolitis-associated interstitial lung disease (RB-ILD). Consequently, 1685 IPF patients were enrolled in the current study. In these study subjects, age, sex, diagnostic method, smoking status and amount, pulmonary function test (PFT), high-resolution computed tomography (HRCT) findings, co-morbidities, respiratory symptoms, arterial blood gas analysis (ABGA), and survival were investigated. Chest CT findings were interpreted by specialized thoracic radiologists at each hospital. Greater than 10% of CT findings were recorded as positive. Hospital databases were screened for diagnosis of IPF according to the 2002 ATS/ERS criteria^2^ and were recorded in a web-based registry (www.ild.or.kr).

Diagnoses were confirmed at each hospital by a multidisciplinary team consisting of specialists in pulmonary medicine, radiology, and pathology according to the patients’ dates of birth. Additionally, the members of the Scientific Committee reviewed all the cases regardless of their inclusion.

### Diagnostic Criteria

According to the international consensus classification,^2^ a surgical lung biopsy is required for the definitive diagnosis of IPF. However, a diagnosis of IPF can be considered in the absence of a surgical lung biopsy specimen if certain major and minor criteria are met. In such cases, all 4 major criteria and at least 3 of the 4 minor criteria must be satisfied.^2^ When a biopsy specimen was not available, all the major criteria except the last (transbronchial lung biopsy specimen or bronchoalveolar [BAL] fluid sample showing no features to support an alternative diagnosis) applied optionally, and at least 3 of the 4 minor criteria had to be fulfilled. For patients with a surgical biopsy specimen showing UIP, only the major criteria were considered relevant. Although a surgical lung biopsy is required for accurate diagnosis, a patient who was too old and had a low lung function was diagnosed clinically at the physician's discretion without undergoing a biopsy. Additionally, a patient who refused to undergo the surgical lung biopsy was diagnosed clinically.

### Statistical Analysis

The Student *t* test was used to compare continuous variables, whereas Pearson *χ*^2^ test was used to compare categorical variables. Patients were censored if they were still alive when last contacted (censored at the last status date), or had received a lung transplant (censored at the time of the transplant). Survival time was calculated as the time since diagnosis.

The survival was estimated using the Kaplan–Meier method models. The log-rank statistic was used to compare survival among groups. The effect of each variable on the risk of death after controlling for age, sex, and pulmonary function (GAP predictive variables) was modelled using the Cox proportional hazards regression.^16^

Unless otherwise noted, all tests were 2-sided and performed at the 0.05 significance level. SPSS Version 20 (SPSS, Chicago, IL) was used for all analyses.

### Ethics Statement

This study protocol was reviewed and approved by the Institutional Review Board of the Severance Hospital Ethics Committee (IRB approval number: 4–2009–0372), which deemed that informed consents were waived.

## RESULTS

### Demographic Characteristics

A total of 1685 patients with IPF (cIPF: 1027 and sIPF: 658) were enrolled in this study. The mean follow-up duration was 17.7 ± 15.7 months. The demographic characteristics of the participants are presented in Table 1. The mean age of the participants was 67.9 ± 9.6 years and the mean age was higher in cIPF than in sIPF group (*P* < 0.001). The proportion of males was higher in cIPF than in sIPF group (*P* < 0.001). Regarding the smoking history, the duration and total amount of smoking were different between the 2 groups (*P* < 0.001, respectively). The proportion of nonsmokers was higher in the sIPF group (*P* < 0.001). The GAP index was calculated in this study as Ley et al^16^ suggested in 2012. The cIPF group showed significantly higher GAP indices than those of sIPF group (*P* < 0.001).

**TABLE 1:**
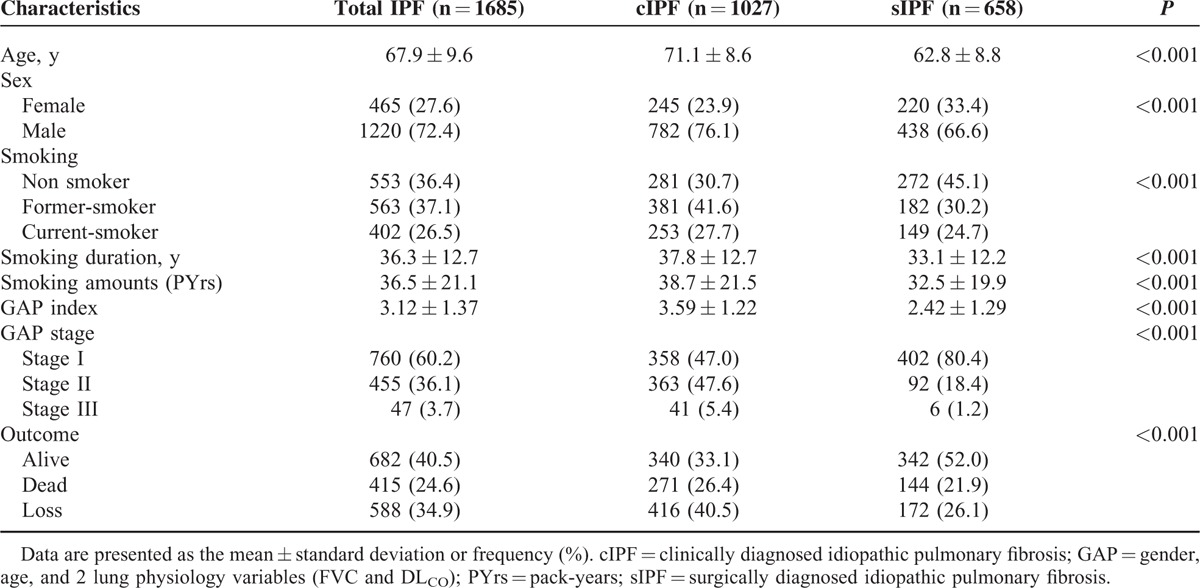
Baseline Characteristics of Patients With cIPF and sIPF

### Clinical Characteristics

Table 2 shows the initial pulmonary function, arterial blood gas analysis (ABGA), and radiologic characteristics of the participants. At presentation, the predicted forced vital capacity (FVC [%]), predicted forced expiratory volume in 1 second (FEV_1_ [%]), and the predicted diffusion capacity of the lungs for carbon monoxide (DL_CO_ [%]) were better in the sIPF patients (*P* < 0.001, respectively). Resting PaO_2_ and PaCO_2_ at presentation were also significantly different between the 2 groups (*P* < 0.001, respectively). Honeycombing change (72.7%) and ground glass opacities (57.2%) were present in most patients, whereas nodular lesions were only present in some patients (20.4%). Ground glass opacities (*P* < 0.001) were more frequently shown in patients with sIPF, whereas the proportion of honeycombing changes was significantly higher in cIPF group (*P* < 0.001).

**TABLE 2:**
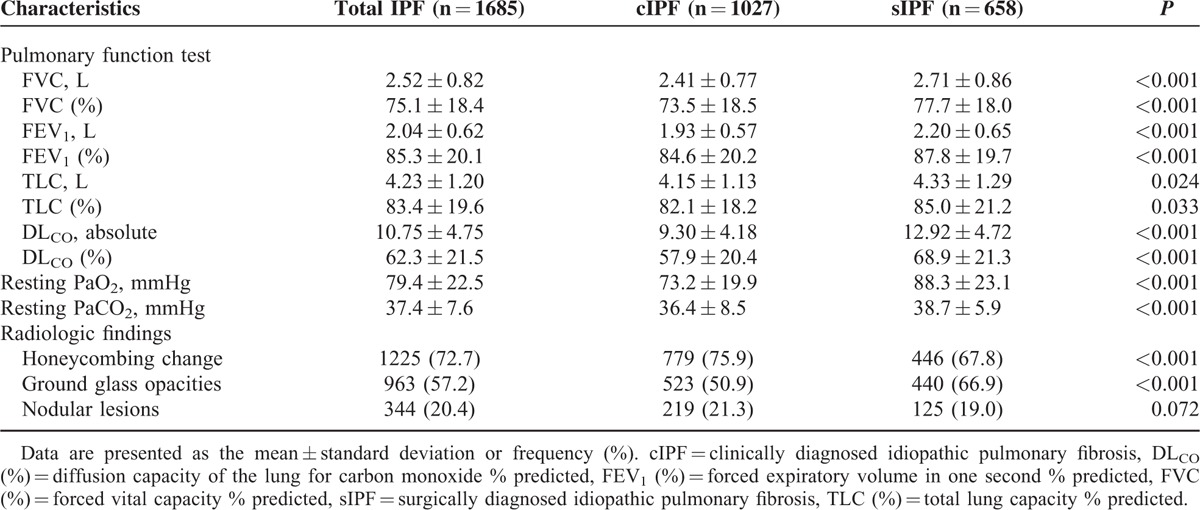
Initial Physiologic and Radiologic Characteristics of Patients with cIPF and sIPF

Table S1 shows the comorbidities of patients. Diabetes and hypertension are the most common comorbidities in both groups. Diabetes, cardiovascular diseases, and chronic renal diseases are significantly more frequent in cIPF than sIPF group.

Initial presenting symptoms at diagnosis are presented in Table 3. The average symptom duration at presentation was 10.9 ± 20.9 months. There was no difference between cIPF and sIPF group. Dyspnea on exertion and cough were the most common symptoms. The 2 groups did not differ with respect to symptoms.

**TABLE 3:**
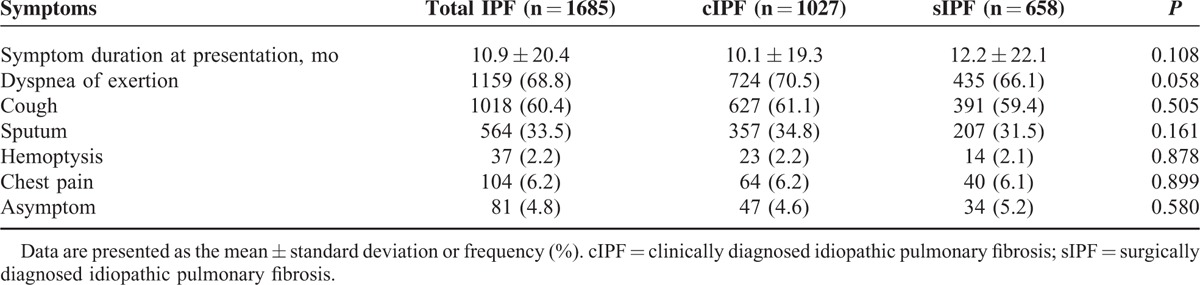
Initial Presenting Symptoms of cIPF and sIPF

### Clinical Factors Associated With Survival

**FIGURE 2:**
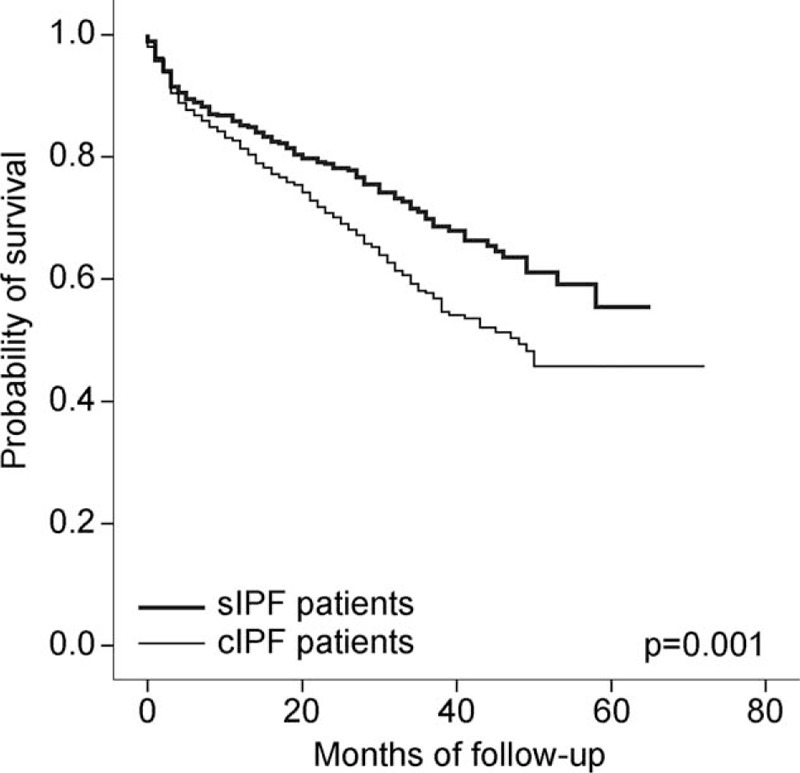
Kaplan–Meier estimates of survival for patients with IPF according to diagnostic method. cIPF = clinically diagnosed idiopathic pulmonary fibrosis, sIPF = surgically diagnosed idiopathic pulmonary fibrosis.

**FIGURE 3:**
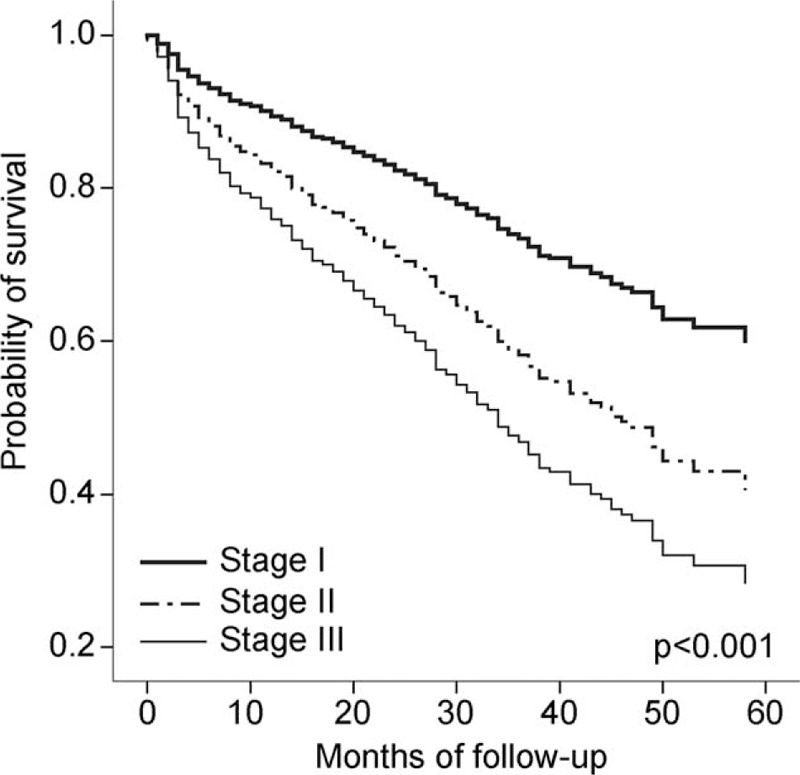
Survival analysis for total IPF patients according to GAP stage with Cox proportional hazard model. GAP = gender, age, and 2 lung physiology variables (FVC and DL_CO_).

The clinical factors associated with survival are shown in Table 4 (univariate analysis), Table 5 (multivariate analysis), Table S2, and Figures 2 and 3. In the Kaplan–Meier survival analysis, patients with cIPF showed a significantly poor prognosis compared with those with sIPF (Figure 2, *P* = 0.001). Increased age, lower FVC, FEV_1_, TLC, DL_CO_, and PaO_2_, the presence of honeycombing changes on radiological tests, and the presence of combined lung cancer were associated with a poor prognosis in all patients with IPF on univariate analysis (Table 4). However, age was not a significant factor in the cIPF group and the presence of honeycombing change was not significant in either of the cIPF or sIPF groups. Sex, smoking amount, and duration of the symptoms were not significantly related to mortality.

**TABLE 4:**
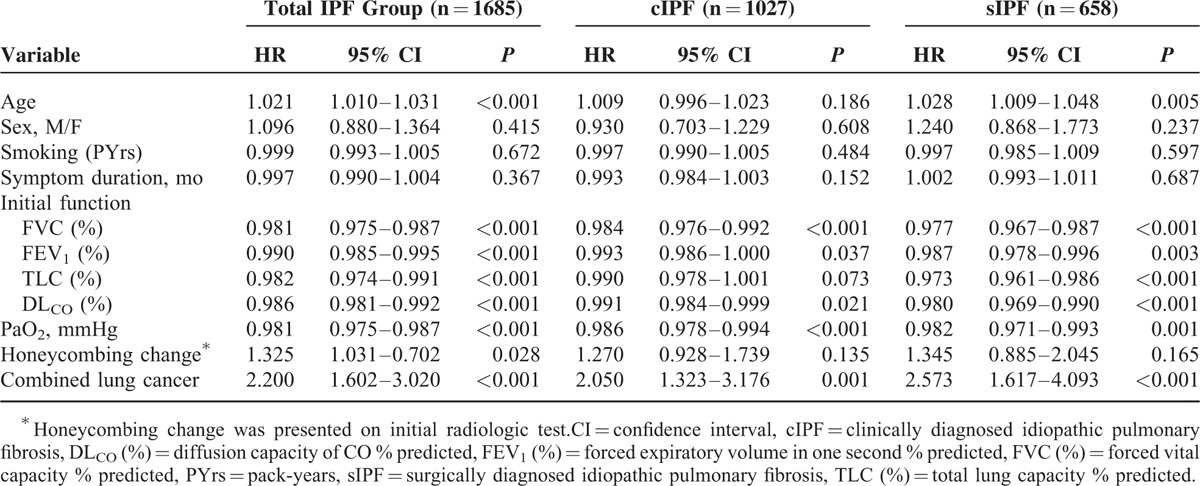
Clinical Factors Associated With Survival in cIPF and sIPF (Univariate Analysis)

**TABLE 5:**
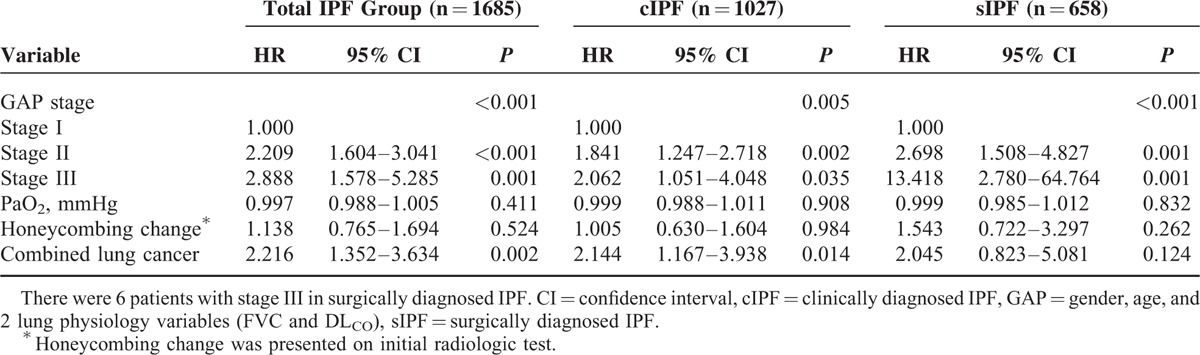
Clinical Factors Associated With Survival in cIPF and sIPF (Multivariate Analysis)

**FIGURE 4:**
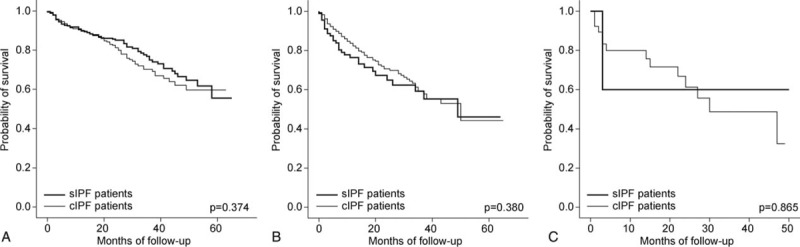
Survival analysis for IPF group according to GAP stage: (A) GAP stage I (n = 760), (B) GAP stage II (n = 455), (C) GAP stage III (n = 47). cIPF = clinically diagnosed idiopathic pulmonary fibrosis, sIPF = surgically diagnosed idiopathic pulmonary fibrosis, GAP = gender, age, and 2 lung physiology variables (FVC and DL_CO_). There are only 6 patients in the surgically diagnosed IPF group with GAP stage III.

In analyses using GAP stage, an advanced GAP stage–except stage III in sIPF (*P* = 0.215, n = 6)—was associated with a significantly poor prognosis in all subjects in both the cIPF and sIPF groups (Table S2 and Figure 3). However, there was no significant difference between the two groups after adjusting for GAP stage (Figure 4).

In a multivariate analysis, GAP stage was an independent prognostic factor in both groups, but PaO_2_ and honeycombing change were not (Table 5). Furthermore, combined lung cancer was significantly associated a poor outcome in the total IPF group and the group with cIPF (*P* = 0.002 and *P* = 0.014, respectively), but not in the sIPF group (*P* = 0.124).

The treatment history of patients is demonstrated in Table S3. The proportion of patients treated with an immunosuppressant was higher in the sIPF than the cIPF. Only 1 patient with sIPF underwent lung transplantation. Respiratory failure (45.1%) and infection (34.1%) were the most common causes of death (Table 6). The proportion of patients who experienced respiratory failure was higher in the cIPF than in the sIPF (*P* = 0.035).

**TABLE 6:**
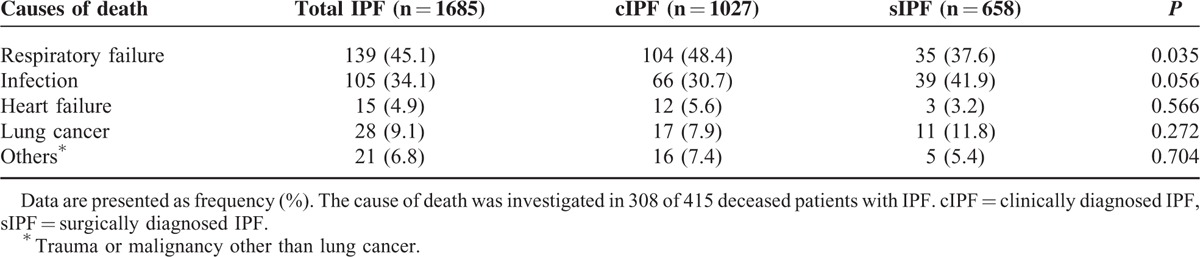
Cause of Death in cIPF and sIPF

## DISCUSSION

This study demonstrated that GAP staging was applicable to both the sIPF and cIPF groups that had similar mortality predictions according to GAP stage.

Since Ley et al^16^ reported the GAP index and staging system in 2012, it has simply been used for predicting the clinical course of patients. However, the original cohort included patients diagnosed by both surgical and clinical methods; therefore, the GAP model has never been stipulated as applicable only to patients diagnosed by biopsy or clinically. The results of the present study show that the predicted prognosis of patients with sIPF is not different from those with cIPF when using the GAP model.

The new 2011 ATS/ERS IPF diagnostic criteria have reduced the importance of surgical lung biopsy; however, HRCT has become essential. As a result, physicians now more frequently diagnose IPF without a lung biopsy.^3^ However, the prognosis of patients with IPF who are diagnosed by surgery is unknown. This study aimed to evaluate whether sIPF and cIPF groups have different characteristics, as well as the predictability of the GAP staging system in patients with IPF who eventually underwent surgical lung biopsy in comparison with patients with cIPF. Although there may be sampling errors and interobserver variations in the surgical lung biopsy procedure, lung biopsy still plays an important role in diagnosing IPF, especially when the HRCT and/or clinical features are uncertain to make a diagnosis.^17^ Because of the different prognoses and therapies, an accurate diagnosis among the interstitial lung diseases is very important.^18^ It is also important to allow for the investigation of potentially different mechanisms that may be operative during the early, intermediate, and end stages of the disease. This knowledge could lead to the implementation of targeted therapeutic interventions during the early disease stages.

Raghu et al^19^ reported that patients with new-onset IPF could be diagnosed by a clinical or radiological expert's assessment; however, approximately one-third of patients with new-onset IPF needed surgical lung biopsy for accurate diagnosis despite an expert review of clinical-radiological features. Additionally, another study demonstrated that 44 patients were described as having possible nonspecific interstitial pneumonia (NSIP) or definite NSIP by HRCT findings. Ultimately, 26 (59.1%) of those 44 patients were diagnosed with IPF, whereas the remaining 18 patients (40.9%) were diagnosed with NSIP by histopathology.^20^ Therefore, a lung biopsy may be required when a diagnosis is uncertain, when less experienced clinicians manage patients, and when the clinical diagnosis is not IPF.^21^

Similarly, previous studies^22–25^ have shown that HRCT findings in 30% to 50% of patients with IPF are not typical for the IPF criteria. Typical CT findings of IPF included the following: subpleural, basal predominance; reticular abnormality; honeycombing with or without traction bronchiectasis; and absence of features listed as inconsistent with the UIP pattern. Consistent with the results of these studies, the proportion of patients with sIPF was 39.1% in our study. Furthermore, the proportion of honeycombing findings was significantly lower in the sIPF group (*P* < 0.001).

Generally, physicians are reluctant to diagnose IPF by surgical lung biopsy if patients are clinically unsuitable for surgery. As a result, patients with cIPF tend to be older and male. Ley et al^16^ suggested male sex as one of the poor prognostic factors. Patients with cIPF also had a more severe smoking history, worse lung function results, relatively more dominant honeycombing on HRCT, and more comorbidities. This cIPF group tended to undergo conservative care rather than aggressive treatment. In general, owing to the worse clinical features among patients with cIPF, they showed a significantly poorer prognosis than patients with sIPF in a Kaplan–Meier survival analysis (*P* = 0.001). Although the patients had the same diagnosis, the physician's selection bias led to the differences in clinical features reported and the mortality in patients with cIPF and sIPF. We expected that cIPF group would demonstrate a relatively longer duration of symptoms and a higher frequency of the following symptoms: dyspnea, cough, sputum, hemoptysis, and chest pain. However, there was no significant difference between the cIPF and sIPF groups regarding symptoms. This may be one reason why the severity of IPF varies according to the subjective perception of symptoms and the healthcare provider's awareness.^3^

Ley et al^16^ demonstrated the GAP index and staging system for predicting mortality in mixed groups of clinically and surgically diagnosed IPF. As in their study, our study showed that an advanced GAP stage tended to be associated with a significantly worse prognosis. Previous studies have shown that patients with IPF who had discordant UIP (histological UIP pattern and nontypical CT pattern), which is usually diagnosed surgically, had a better prognosis than in patients with IPF who had concordant UIP (histological UIP and typical CT pattern), which is usually diagnosed clinically.^20,25^ However, these studies had insufficient sample sizes to compare patient prognoses, and they did not consider individual clinical conditions. Similar to previous studies, ours showed that patients with sIPF had a better prognosis than those with cIPF, as assessed by a Kaplan–Meier analysis. However, there were no significant differences in mortality between patients with the same GAP stage, regardless of the diagnostic method used. Therefore, this study shows that the GAP model is applicable in predicting the prognosis of both sIPF and cIPF groups.

Furthermore, our univariate analysis showed that PaO_2_ and honeycomb findings on HRCT, and combined lung cancer could be prognostic factors in patients with IPF (Table 4). Previous studies have shown that hypoxemia and a quantitative scoring system by HRCT could be useful in predicting the prognosis of IPF patients.^26–29^ However, a multivariate analysis revealed that PaO_2_ and honeycomb findings were not significant in our study. These results might be explained by the fact that we were unable to examine lung fibrosis scores and emphysema grades in CT findings and did not evaluate physiological function using test like 6-minute-walk distance (6MWD).

This study has some limitations. First, we used a definition from the 2002 ATS/ERS guidelines, which were updated in 2011. In the past, surgical lung biopsy had an important role in IPF diagnoses, whereas HRCT has an essential role in the updated guideline. Additionally, efficacy-proven drugs (pirfenidone or nintedanib) were not used in the past. Although Ley et al^16^ also created the GAP model using a derivation cohort and validation cohort that had been diagnosed between 2000 and 2010, there may be some differences between this study population's patients and those diagnosed by the 2011 ATS/ERS guidelines. Second, the duration of follow-up for this study's population was relatively short, with an expected median survival time of approximately 3 years in patients with IPF. However, this was an adequately large, multicenter study for validating the GAP model and, in the original article by Ley et al, the median follow-up time was also not relatively long (1.7 years in the derivation cohort), Third, chronic hypersensitivity pneumonitis is difficult to distinguish from IPF. However, we tried to identify patients with chronic hypersensitivity pneumonitis by history, clinical presentation, and laboratory results. The Scientific Committee of the Korean Academy of Tuberculosis and Respiratory Diseases also reviewed the diagnoses. Finally, our study had only 6 sIPF patients with a GAP stage of III, and the prognosis survival analysis of this group was not significant. This may be because we did not check the “cannot perform” category of DL_CO_.

In conclusion, patients with sIPF showed better clinical features than patients with cIPF. Moreover, sIPF patients are found to be younger and have favorable lung function when compared with cIPF patients. The GAP model could be applicable in the prediction of prognosis in both sIPF and cIPF groups. In addition, IPF patients might have the same prognosis after adjusting for GAP stage regardless of the diagnostic method used.

## Supplementary Material

Supplemental Digital Content
