# Supplementary material for: Comparisons of Prognosis between Surgically and Clinically Diagnosed Idiopathic Pulmonary Fibrosis Using Gap Model: A Korean National Cohort Study
Source: Medicine (Baltimore). 2016 Mar 18;95(11):e3105. doi: 10.1097/MD.0000000000003105 (PMC4839935; doi:10.1097/MD.0000000000003105)
Supplement: Supplemental Digital Content [file medi-95-e3105-s001.doc]

**Table S1.** Co-morbidities of clinically and surgically diagnosed idiopathic pulmonary fibrosis patients

| Co-morbidities | Total IPF  (n=1,685) | cIPF  (n=1,027) | sIPF  (n=658) | p-value |
| --- | --- | --- | --- | --- |
| History of pulmonary TB | 204 (12.1) | 119 (11.6) | 85 (12.9) | 0.679 |
| Diabetes mellitus | 300 (17.8) | 198 (19.3) | 102 (15.5) | 0.048 |
| Hypertension | 368 (21.8) | 225 (21.9) | 143 (21.7) | 0.932 |
| Cardiovascular diseases | 104 (6.2) | 80 (7.8) | 24 (3.7) | 0.001 |
| Cerebrovascular diseases | 37 (2.2) | 27 (2.6) | 10 (1.5) | 0.130 |
| Chronic liver diseases | 36 (2.1) | 22 (2.1) | 14 (2.1) | 0.984 |
| Allergic diseases | 34 (2.1) | 20 (2.0) | 14 (2.1) | 0.797 |
| Chronic renal diseases | 25 (1.5) | 21 (2.0) | 4 (0.6) | 0.017 |
| Chronic lung diseases | 30 (1.8) | 23 (2.2) | 7 (1.1) | 0.075 |
| Lung cancer | 111 (6.6) | 59 (5.7) | 52 (7.9) | 0.082 |
| Other malignancies | 77 (4.6) | 49 (4.8) | 28 (4.3) | 0.621 |

Data are presented as frequency (%).

cIPF, clinically diagnosed IPF; sIPF, surgically diagnosed IPF; TB, tuberculosis

Table S2. Survival analysis according to GAP stage in clinically and surgically diagnosed patients with idiopathic pulmonary fibrosis by Cox proportional hazard models

| Variable | Total IPF group (n=1,685) | | | cIPF (n=1,027) | | | sIPF (n=658) | | |
| --- | --- | --- | --- | --- | --- | --- | --- | --- | --- |
|  | HR | 95% CI | p-value | HR | 95% CI | p-value | HR | 95% CI | p-value |
| GAP stage |  |  | <0.001 |  |  | 0.003 |  |  | 0.001 |
| Stage I | 1.000 |  |  | 1.000 |  |  | 1.000 |  |  |
| Stage II | 1.750 | 1.369-2.239 | <0.001 | 1.558 | 1.135-2.104 | 0.006 | 2.144 | 1.398-3.288 | <0.001 |
| Stage III | 2.450 | 1.460-4.112 | 0.001 | 2.304 | 1.295-4.099 | 0.005 | 2.435 | 0.596-9.943 | 0.215 |

There were six patients with stage III in surgically diagnosed IPF.

cIPF, clinically diagnosed idiopathic pulmonary fibrosis; sIPF, surgically diagnosed idiopathic pulmonary fibrosis; GAP, gender, age, and two lung physiology variables (FVC and DL_CO_); CI, confidence interval

**Table S3.** Treatment modalities in clinically and surgically diagnosed patients with idiopathic pulmonary fibrosis (IPF)

| Treatment | Total IPF  (n=1,685) | cIPF  (n=1,027) | sIPF  (n=658) | p-value |
| --- | --- | --- | --- | --- |
| Conservative care | 633 (41.0) | 448 (46.9) | 185 (31.4) | <0.001 |
| PL ± NAC/Col | 462 (29.9) | 283 (29.6) | 179 (30.4) | 0.752 |
| PL plus Aza/CYC ± NAC/Col | 268 (17.4) | 131 (13.7) | 137 (23.3) | <0.001 |
| Aza/CYC ± NAC/Col | 61 (4.0) | 17 (1.8) | 44 (7.5) | <0.001 |
| NAC/Col | 119 (7.7) | 76 (8.0) | 43 (7.3) | 0.695 |
| Lung transplantation | - | - | 1 (0.2) | 0.381 |

Data are presented as frequency (%).

cIPF, clinically diagnosed IPF; sIPF, surgically diagnosed IPF; PL, prednisone; Col, colchicine; NAC, N-Acetlycysteine; Aza, azathioprine; CYC, cyclophosphamide
